# Success

**Published:** 1907-05

**Authors:** 


### SUCCESS.

It is not the man who accentuates obstacles or who invariably anticipates failure that we are patient with or attracted by; not the man who views every suggestion as an attempt to impoverish him; who recoils from the hearty salutation or the warm hand clasp; who seeks lurking in every act of others evidences of a design to attack the purse; who sees proof of an unholy alliance when those of opposite se xare cordial to one another. Such a man is a misanthrope, a pessimist, and sooner or later will become a hypochondriac, a misfit in all his relations with others. He will succeed but measurably if at all, and walk alone because it is next to impossible to have any pleasure in his company; he is—a joy killer. The man who succeeds is he that views the pathway of life with the golden hours of probability; who regards the rungs of the ladder by which he ascended as veritable jewels, reflecting hope and lighting the way to the temple of achievement. He may be visionary; seem unsubstantial, altogether impracticable, but he is sure to believe in himself and in his fellowmen; he will see the opportunities offered, will meet them more than half way, will love his wife and children, count the price of nothing that will add to their comfort or happiness; will have friends who sooner or later will take him at his own valuation, which is sure to be high. This is the man who wears the epaulets, enjoys public esteem and whose check is never protested; one who succeeds; who is an optimist.—Editorial, Dietic and Hygienic Gazette, April, 1907.

